# Supplementary material for: Seasonal variability of ocean circulation near the Dotson Ice Shelf, Antarctica
Source: Nat Commun. 2022 Mar 3;13:1138. doi: 10.1038/s41467-022-28751-5 (PMC8894431; doi:10.1038/s41467-022-28751-5)
Supplement: Supplementary file 1 — Supplementary Information [file 41467_2022_28751_MOESM1_ESM.pdf]

Supplementary Information

Seasonal variability of ocean circulation near the Dotson Ice Shelf, Antarctica

Yang et al.

Supplementary Table 1 | Mooring details

Station	Depth	Latitude Longitude	Observation Period (MM/DD/YYYY)	Instruments	Reference
K4	785 m	74° 10.576' S 112° 8.083' W	01/08/2014 – 01/19/2016	150 kHz ADCP	Upward-looking at 610 m
			01/08/2014 – 01/14/2015	300 kHz ADCP	Downward-looking at 612 m
			01/08/2014 – 12/22/2015	SBE37-SMP-ODO	249 m
			01/08/2014 – 01/19/2016	SBE37-SM	314 m
			01/08/2014 – 01/19/2016	SBE37-SM	385 m
			01/08/2014 – 01/19/2016	SBE37-SM	457 m
			01/08/2014 – 01/19/2016	SBE37-SMP-ODO	527 m
			01/08/2014 – 01/19/2016	SBE37-SM	598 m
			01/08/2014 – 01/19/2016	SBE37-SM	675 m
			01/08/2014 – 01/19/2016	SBE37-SM	747 m
K3	1028 m	74° 10.292' S 112° 31.699' W	01/09/2014 – 01/11/2015	75 kHz ADCP	Upward-looking at 905 m
			01/09/2014 – 01/02/2016	300 kHz ADCP	Downward-looking at 907 m
			01/09/2014 – 01/19/2016	SBE37-SM	247 m
			01/09/2014 – 01/19/2016	SBE37-SM	317 m
			01/09/2014 – 01/19/2016	SBE37-SM	388 m
			01/09/2014 – 01/19/2016	SBE37-SM	468 m
			01/09/2014 – 01/19/2016	SBE37-SM	539 m
			01/09/2014 – 01/19/2016	SBE37-SM	610 m
			01/09/2014 – 01/19/2016	SBE37-SM	682 m
			01/09/2014 – 01/19/2016	SBE37-SM	752 m
			01/09/2014 – 01/19/2016	SBE37-SM	823 m
			01/09/2014 – 01/19/2016	SBE37-SM	894 m
			01/09/2014 – 01/19/2016	SBE37-SM	955 m
			01/09/2014 – 01/19/2016	SBE37-SM	1015 m
K5	774 m	74° 10.946' S 113° 3.823' W	01/09/2014 – 01/20/2016	150 kHz ADCP	Upward-looking at 625 m
			01/09/2014 – 12/24/2015	300 kHz ADCP	Downward-looking at 627 m
			01/09/2014 – 01/20/2016	SBE37-SMP-ODO	272 m
			01/09/2014 – 01/20/2016	SBE37-SM	342 m
			01/09/2014 – 01/20/2016	SBE37-SM	412 m
			01/09/2014 – 01/20/2016	SBE37-SMP-ODO	483 m
			01/09/2014 – 01/20/2016	SBE37-SM	553 m
			01/09/2014 – 01/20/2016	SBE37-SM	622 m
			01/09/2014 – 01/20/2016	SBE37-SM	695 m
			01/09/2014 – 01/20/2016	SBE37-SM	768 m

High resolution mooring systems deployed on January 8–9, 2014 and recovered on January 19–20, 2016 at three locations: K3 (trough center), K4 (east), and K5 (west) to measure the temporal variability and properties of mCDW near the Dotson Ice Shelf. All moorings used dual-ADCP (Acoustic Doppler Current Profilers) consisting of upward-looking 150 kHz (K4 and K5) or 75 kHz (K3) and downward-looking 300 kHz units, along with 8 (K4 and K5) or 12 (K3) Sea-Bird Electronic (SBE) 37-SM or 37-SMP-ODO MicroCATs.

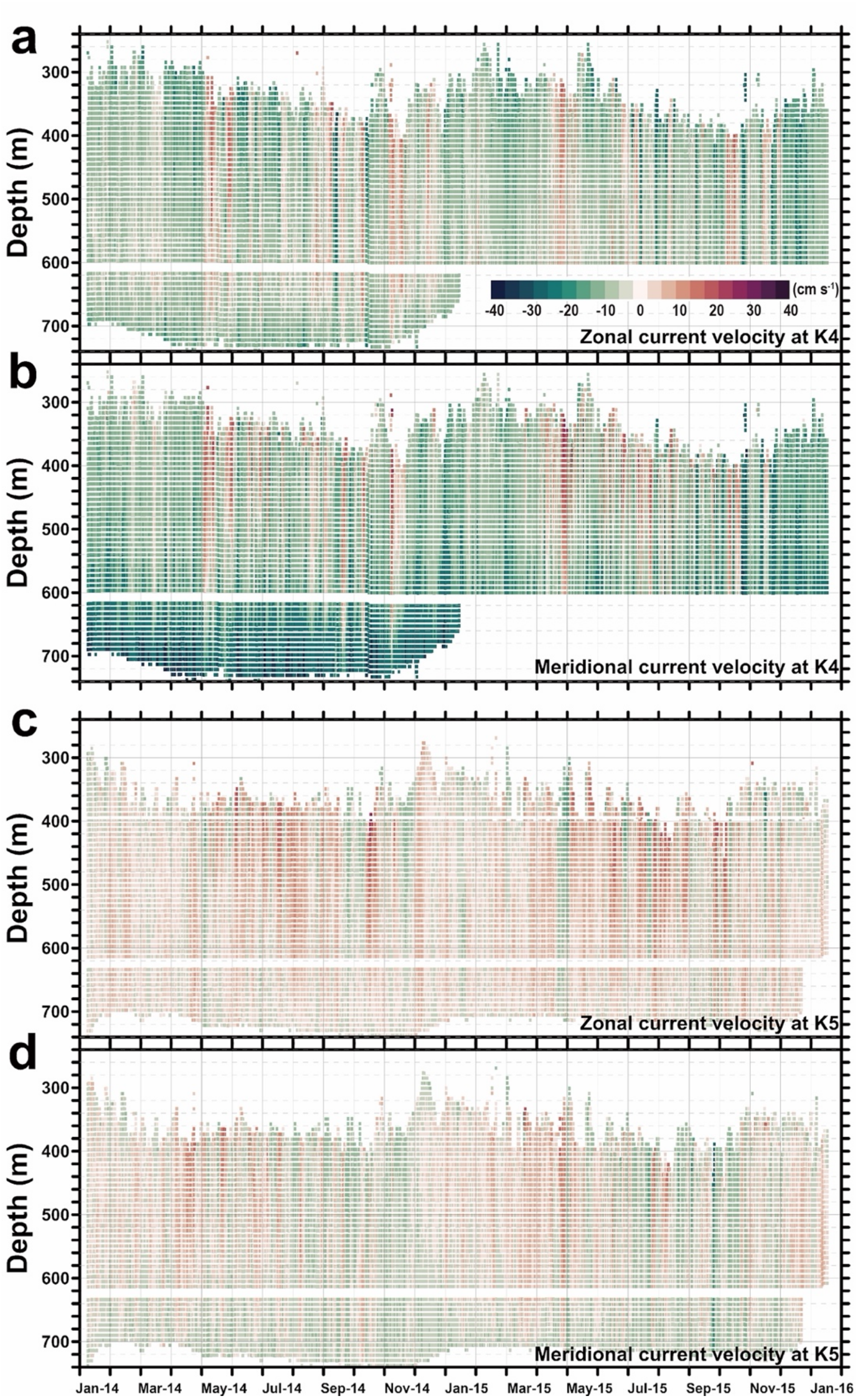

Supplementary Figure 1 | Daily average current velocities at K4 and K5, measured with dual-ADCP (Acoustic Doppler Current Profiler) systems with upward-looking 150 kHz and downward-looking 300 kHz units. (a & b) Zonal and meridional current velocities at mooring K4. **(c & d)** Zonal and meridional current velocities at mooring K5.

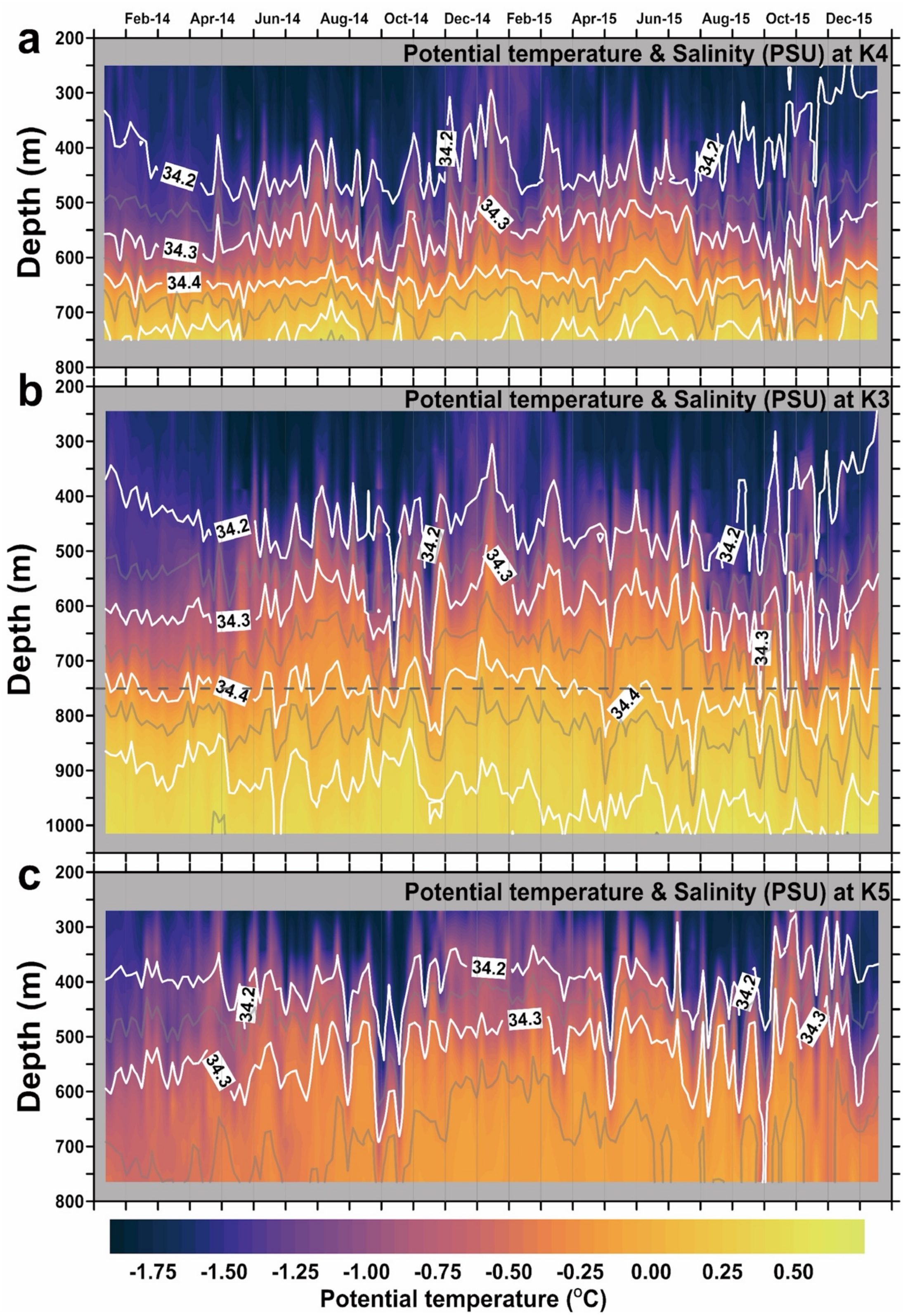

Supplementary Figure 2 | Time series of temperature and salinity at (a) K4, **(b)** K3, and **(c)** K5. Temperature (color) and salinity (white line) were measured by Sea-Bird 37-SM or 37-SMP-ODO MicroCATs from early January 2014 to mid-January 2016.

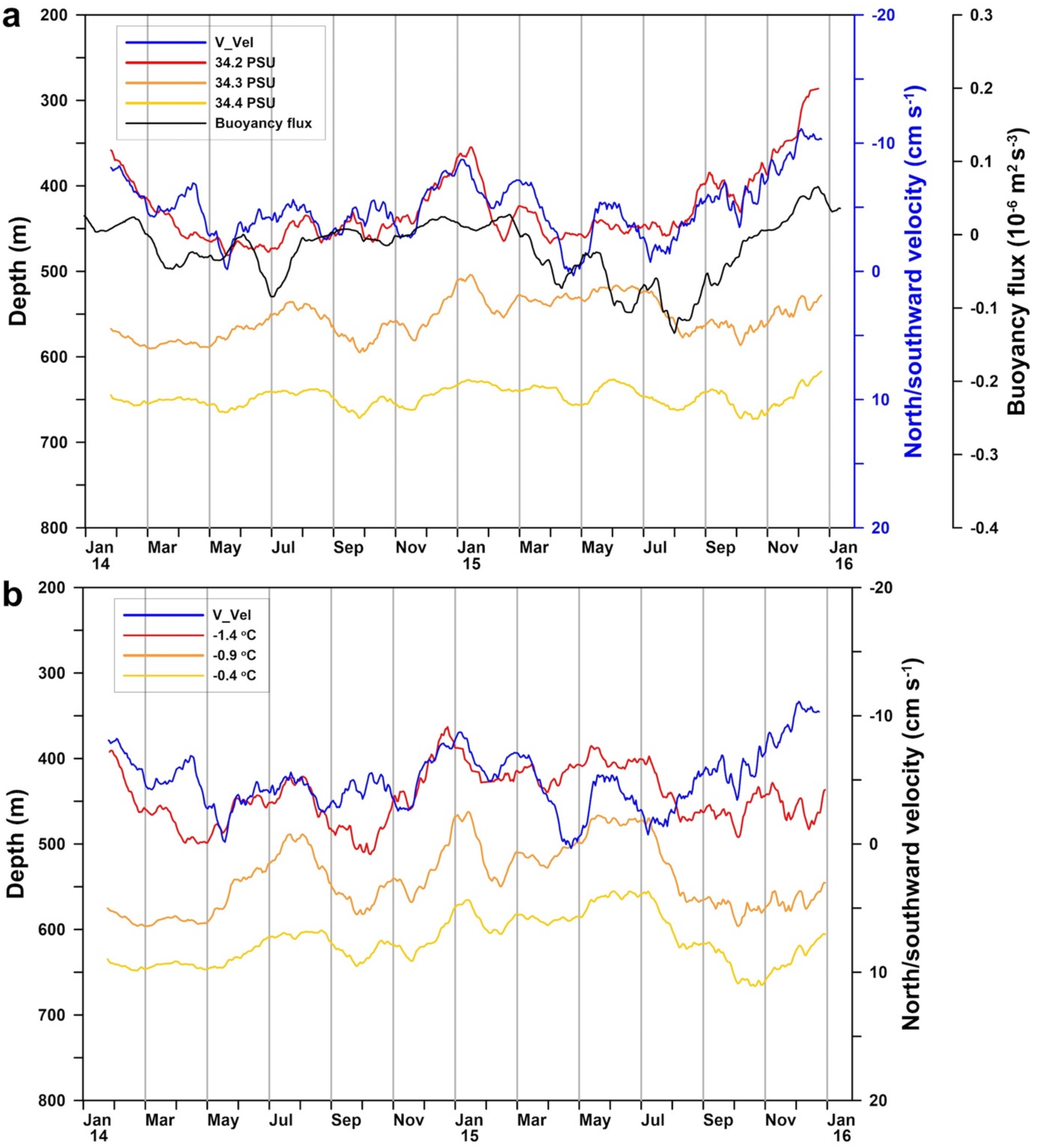

Supplementary Figure 3 | Time series variation of the isohaline and isotherm depth with the meridional velocity at K4 (V_Vel) and Buoyancy flux. (a) 31-day moving average variation of salinity (red, orange, and yellow lines), depth averaged (400 – 600 m) meridional velocity (blue line) and Buoyancy flux (74.125°S, 112.25°W, black line). **(b)** 31-day moving average variation of potential temperature and meridional velocity.

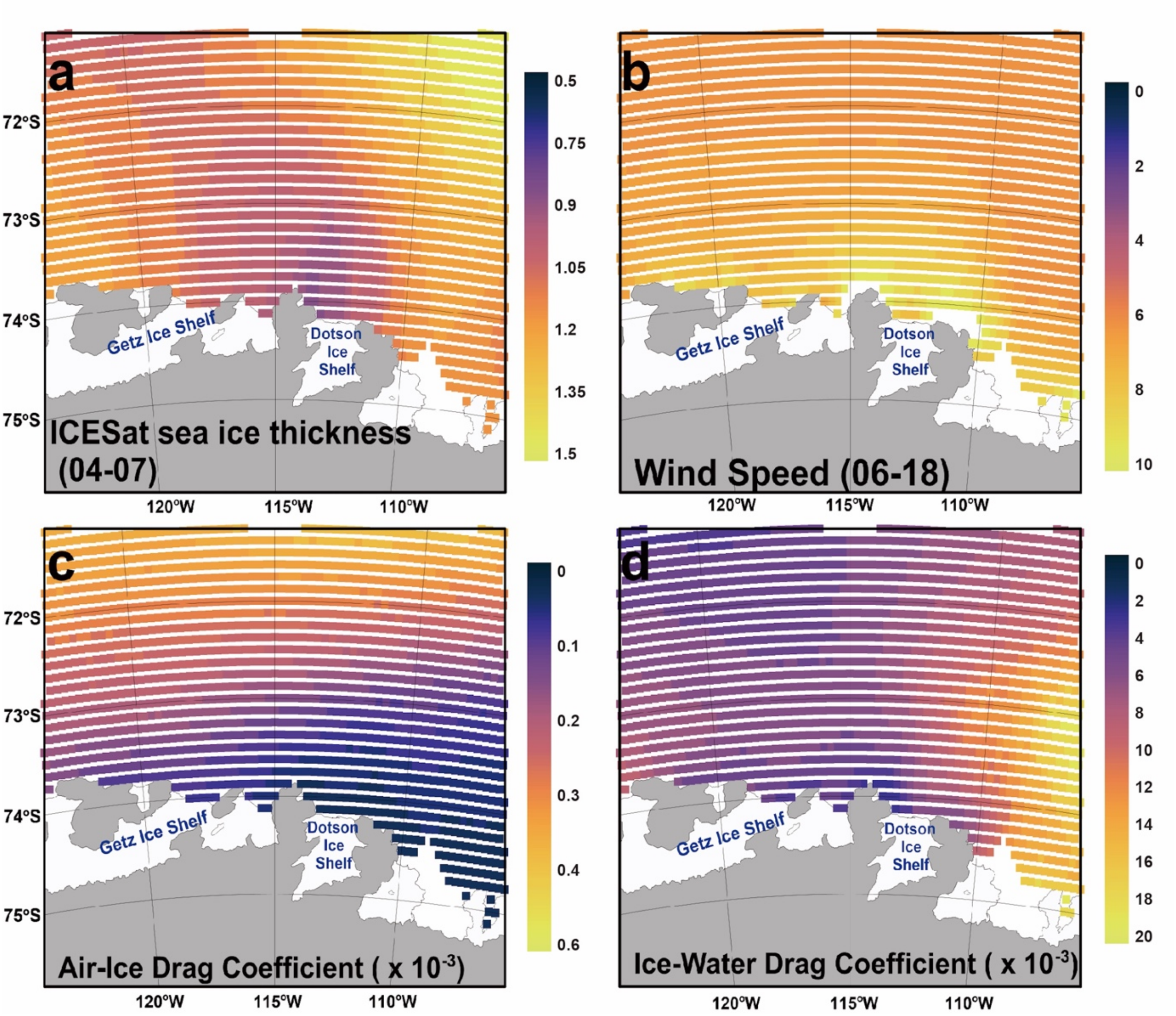

Supplementary Figure 4 | Horizontal distribution of four parameters. (a) Average sea ice thickness during spring and autumn 2004–2007, from ICESat [59,60]. **(b)** Average daily wind speed, 2006–2018, from the Antarctic Mesoscale Prediction System[52]. **(c and d)** Horizontal distributions of air-ice and ice-ocean drag coefficients.

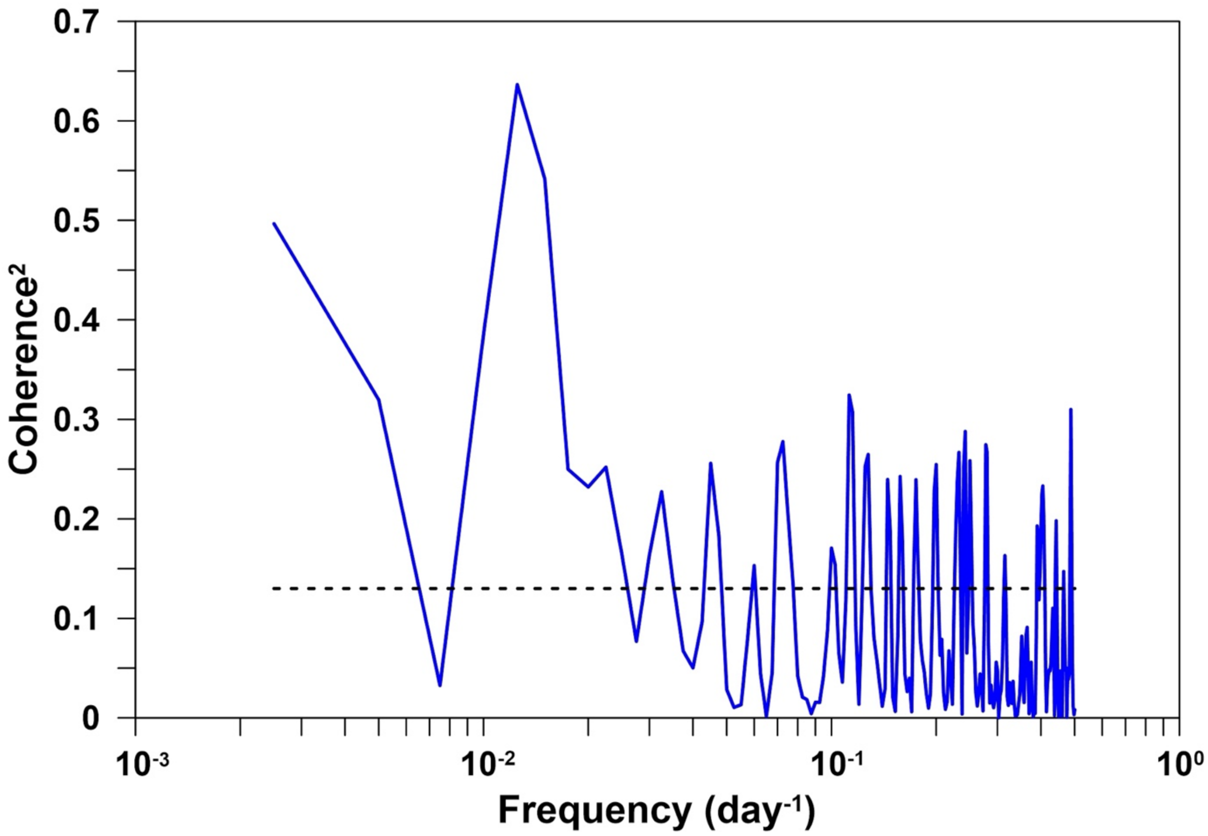

Supplementary Figure 5 | Coherence between the OSSC (Ocean Surface Stress Curl) and the meridional velocity at K4. Coherence between daily mean OSSC (74°S, 112.25 °W) and velocity (mean 400 – 600 m). The dashed line marks the 95% confidence level.

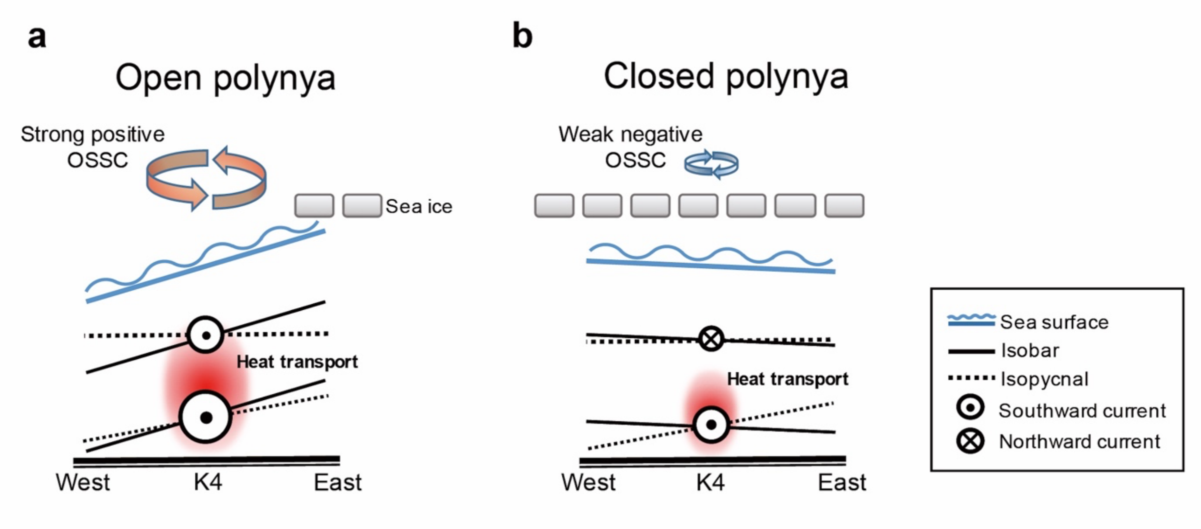

Supplementary Figure 6 | Schematic of processes that the ocean circulation by the interaction between the atmosphere-ocean-ice. (a) In the open polynya season, a strong positive OSSC (Ocean Surface Stress Curl) raises sea levels on the eastern flank, generating barotropic southward flows in the entire water column. In addition, a gradient of isopycnal was steeper with depth due to the mCDW inflow along the eastern slope and strengthened the southward flow near the bottom layer. **(b)** In the closed polynya season, the weakly negative OSSC in the surface lowers the sea level, leading to a weak barotropic northward flow in the entire water column. However, the still strong positive gradient of isopycnal near the bottom generates a southward flow in the bottom layer.

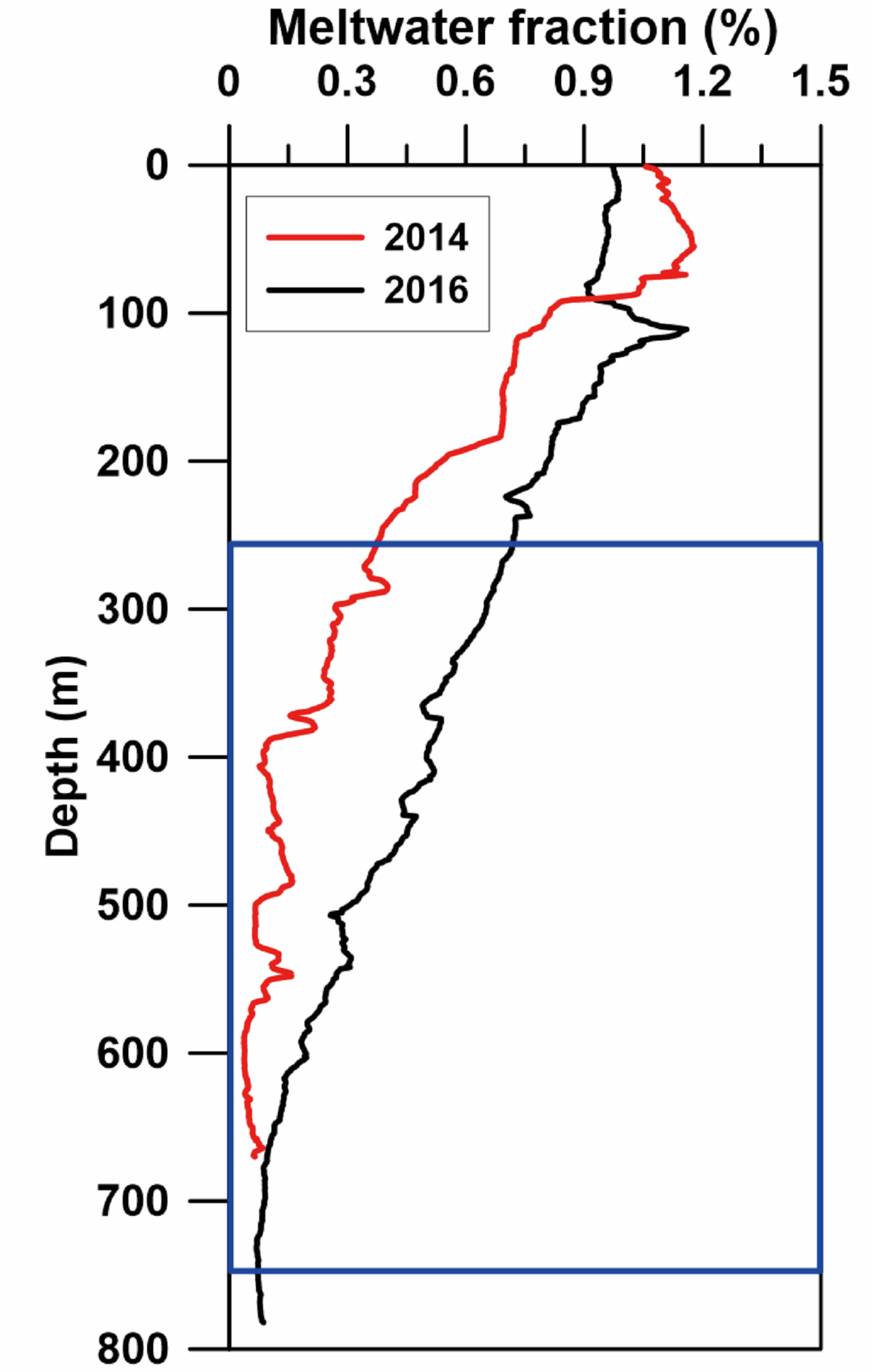

Supplementary Figure 7 | Calculated meltwater fraction from the shipboard CTD data (temperature, salinity, dissolved oxygen) measure in January 2014 and 2016 at the western mooring station (K5). The blue box indicates the covered water column by K5 mooring.

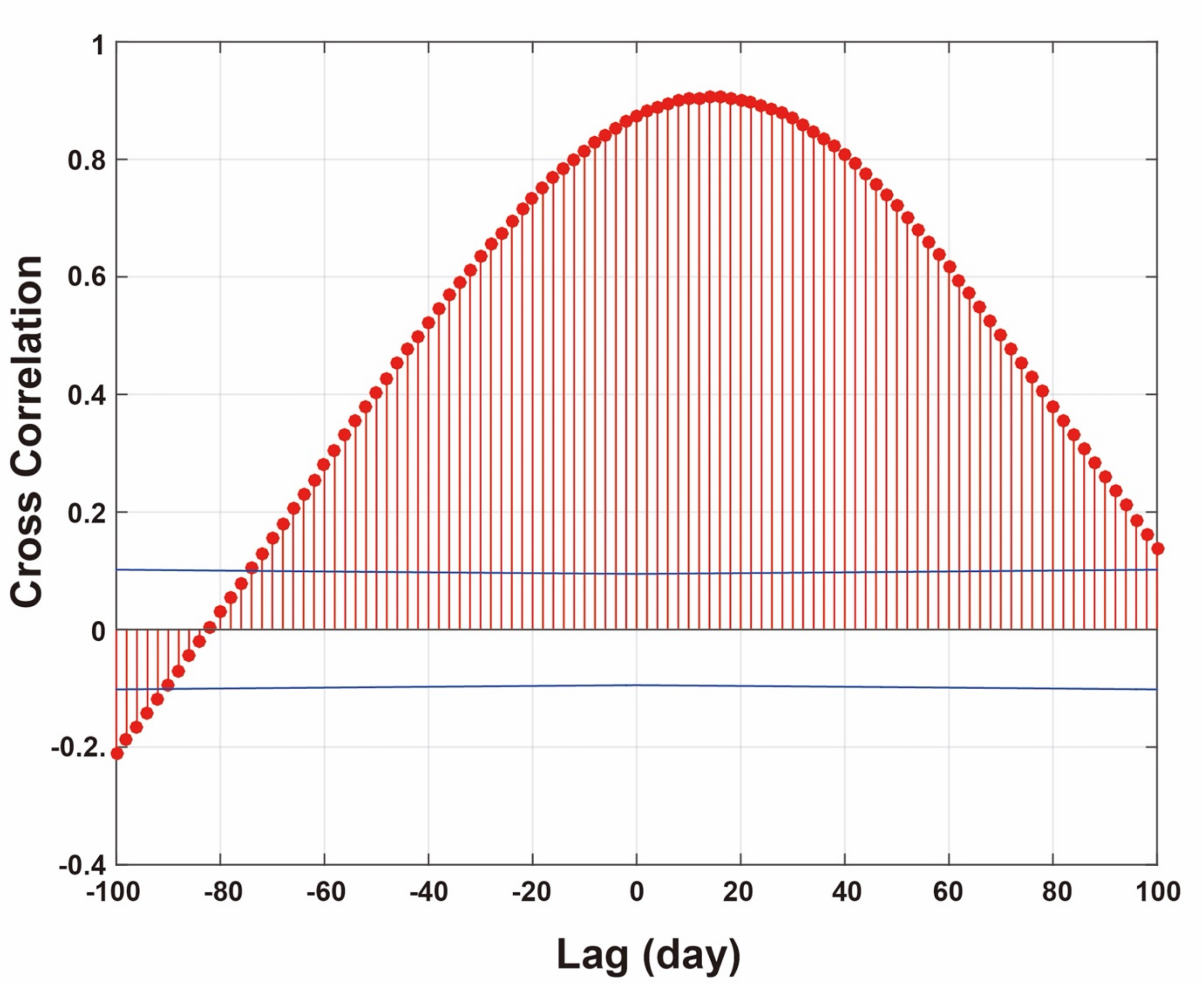

Supplementary Figure 8 | Cross-correlation between meltwater fraction and depth averaged meridional velocity along the western flank. Blue line indicates the 99% confidence interval**.**

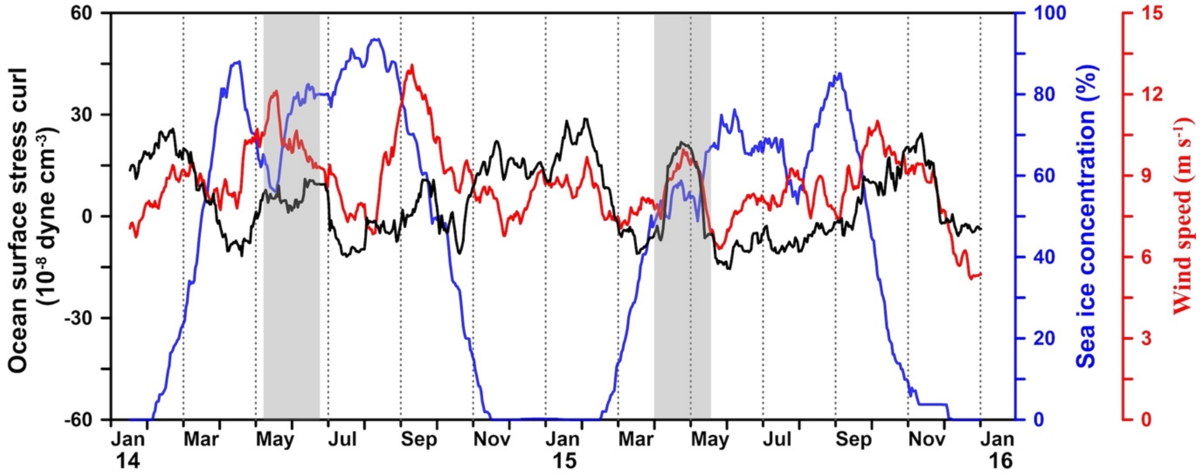

Supplementary Figure 9 | Seasonal variation of OSSC (Ocean Surface Stress Curl), Sea ice concentration and wind speed at 74°S, 112.25°W. Shading is the time of OSSC that coincides with the winter peak of heat transport.

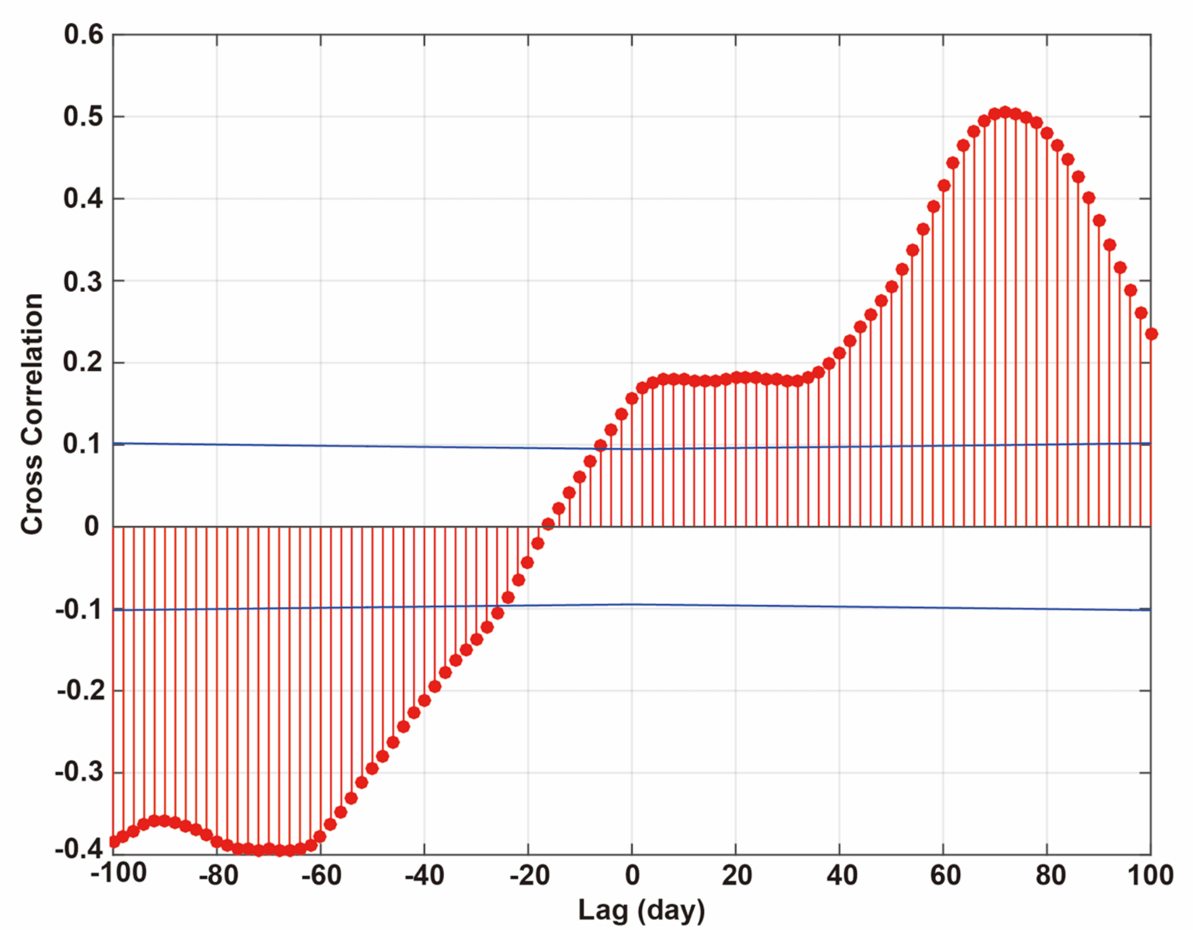

Supplementary Figure 10 | Cross-correlation between Heat transport along the eastern slope and meltwater flux along the western slope. Blue line indicates the 99% confidence interval**.**

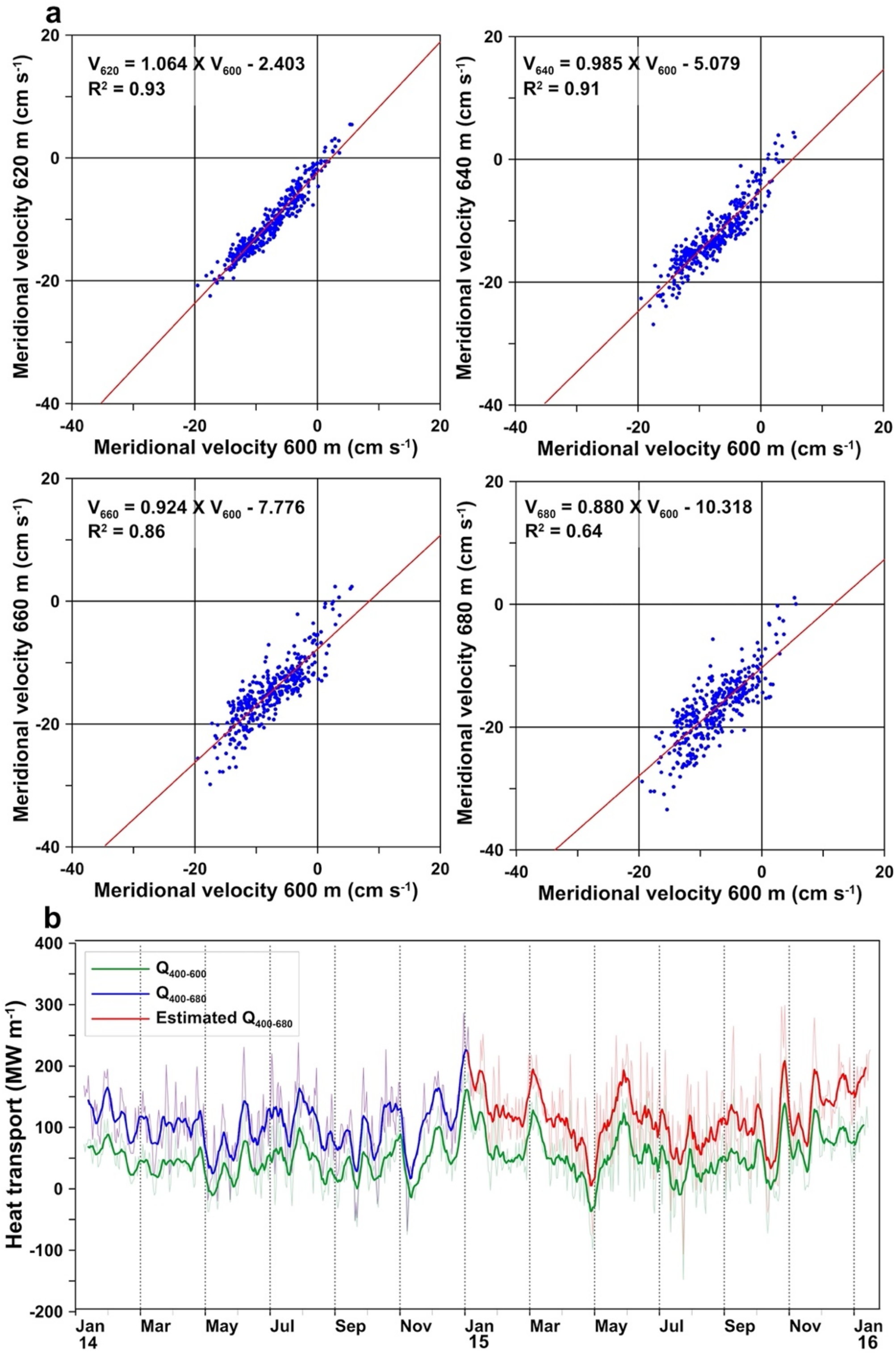

Supplementary Figure 11 | Estimating meridional velocity below 600 m at K4. (a) Regression of velocities between 600 m (V_{600}) and 620 m (V_{620}), 640 m (V_{640}), 660 m (V_{660}), and 680 m (V_{680}). **(b)** Time series of heat transport from 400–600 m ($Q_{400\text{-}600}$) over two years (green) and from 400–680 m ($Q_{400\text{-}680}$) over one year (blue). Heat transport from 400–680 m was extended over two years (red) using the estimated velocity from (a).
